# Degradation versus fibrillogenesis, two alternative pathways modulated by seeds and glycosaminoglycans

**DOI:** 10.1002/pro.4931

**Published:** 2024-02-21

**Authors:** Guglielmo Verona, Sara Raimondi, Diana Canetti, P. Patrizia Mangione, Loredana Marchese, Alessandra Corazza, Francesca Lavatelli, Julian D. Gillmore, Graham W. Taylor, Vittorio Bellotti, Sofia Giorgetti

**Affiliations:** ^1^ Centre for Amyloidosis University College London London UK; ^2^ Department of Molecular Medicine University of Pavia Pavia Italy; ^3^ Research Department Fondazione IRCCS Policlinico San Matteo Pavia Italy; ^4^ Pathology Unit Fondazione IRCCS Policlinico San Matteo Pavia Italy; ^5^ Department of Medicine (DAME) University of Udine Udine Italy; ^6^ Istituto Nazionale Biostrutture e Biosistemi Rome Italy

**Keywords:** amyloidosis, cardiac myopathy, fibrillogenesis, proteolysis, transthyretin

## Abstract

The mechanism that converts native human transthyretin into amyloid fibrils *in vivo* is still a debated and controversial issue. Commonly, non‐physiological conditions of pH, temperature, or organic solvents are used in *in vitro* models of fibrillogenesis of globular proteins. Transthyretin amyloid formation can be achieved under physiological conditions through a mechano‐enzymatic mechanism involving specific serine proteases such as trypsin or plasmin. Here, we investigate S52P and L111M transthyretin variants, both causing a severe form of systemic amyloidosis mostly targeting the heart at a relatively young age with heterogeneous phenotype among patients. Our studies on thermodynamics show that both proteins are significantly less stable than other amyloidogenic variants. However, despite a similar thermodynamic stability, L111M variant seems to have enhanced susceptibility to cleavage and a lower tendency to form fibrils than S52P in the presence of specific proteases and biomechanical forces. Heparin strongly enhances the fibrillogenic capacity of L111M transthyretin, but has no effect on the S52P variant. Fibrillar seeds similarly affect the fibrillogenesis of both proteins, with a stronger effect on the L111M variant. According to our model of mechano‐enzymatic fibrillogenesis, both full‐length and truncated monomers, released after the first cleavage, can enter into fibrillogenesis or degradation pathways. Our findings show that the kinetics of the two processes can be affected by several factors, such as intrinsic amyloidogenicity due to the specific mutations, environmental factors including heparin and fibrillar seeds that significantly accelerate the fibrillogenic pathway.

## 1 INTRODUCTION

**FIGURE 1:**
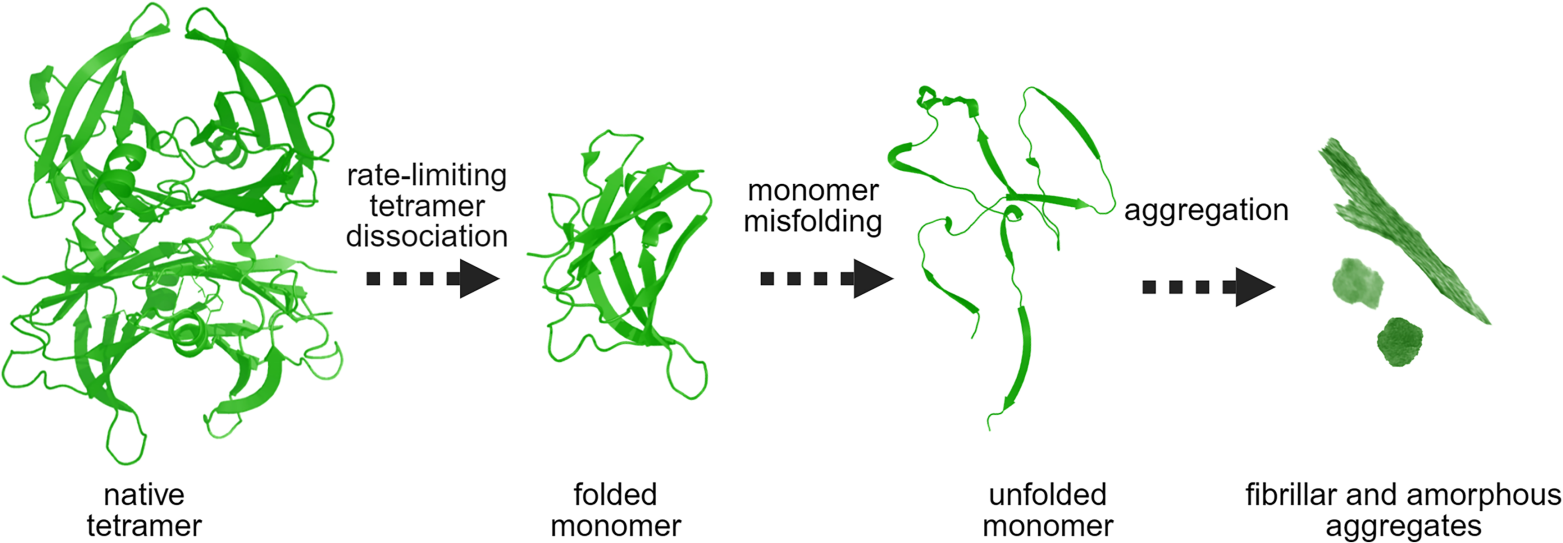
Schematic representation of low pH‐mediated TTR aggregation. Upon exposure to low pH, the TTR tetramer dissociates into dimers and then monomers that are prone to aggregation. Image created with BioRender.com. TTR, transthyretin.

The transition of transthyretin (TTR), from the native tetrameric state to a fibrillar non‐native state represents a fundamental pathogenic event of systemic TTR amyloidosis (ATTR) (Faravelli et al., 2022) yet to define. While the dissociation into monomers is considered the rate‐limiting step for TTR fibril formation (Foss et al., 2005), it is still unknown what actually triggers the process *in vivo*. However, the observation that most of ATTR fibrils consist of a mixture of full‐length and truncated monomers (Ihse et al., 2013) strongly supports the role of proteolysis in ATTR. The residues 49–127 C‐terminal fragment are major component of *ex vivo* TTR amyloid fibrils (Mangione et al., 2014; Thylen et al., 1993) regardless of the presence, nature or position of any amyloidogenic mutation (Bergstrom et al., 2005); other C‐terminal TTR peptides are variably represented (Bergstrom et al., 2005; Hermansen et al., 1995; Ihse et al., 2013). Extensive work has been carried out to recapitulate TTR metamorphism *in vitro*, starting from the early acid‐mediated methods proposed by Colon and Kelly (1992) to more recent observations on the effect of specific proteases under physiological pH, ionic strength and temperature and in combination with shear stress conditions (Klimtchuk et al., 2018; Mangione et al., 2018; Marcoux et al., 2015; Peterle et al., 2020). Although the two proposed mechanisms share the same conclusion that *in vitro* TTR fibrillogenesis requires the disassembly of the native tetramer, the two procedures envisage pathways that are substantially different. The prolonged incubation at low pH, used in the method proposed by Kelly, outlines a two‐state transition in which the conformers populating the transition state are folded and misfolded monomers (Figure 1), all of which are prone to aggregate into fibrils.

**FIGURE 2:**
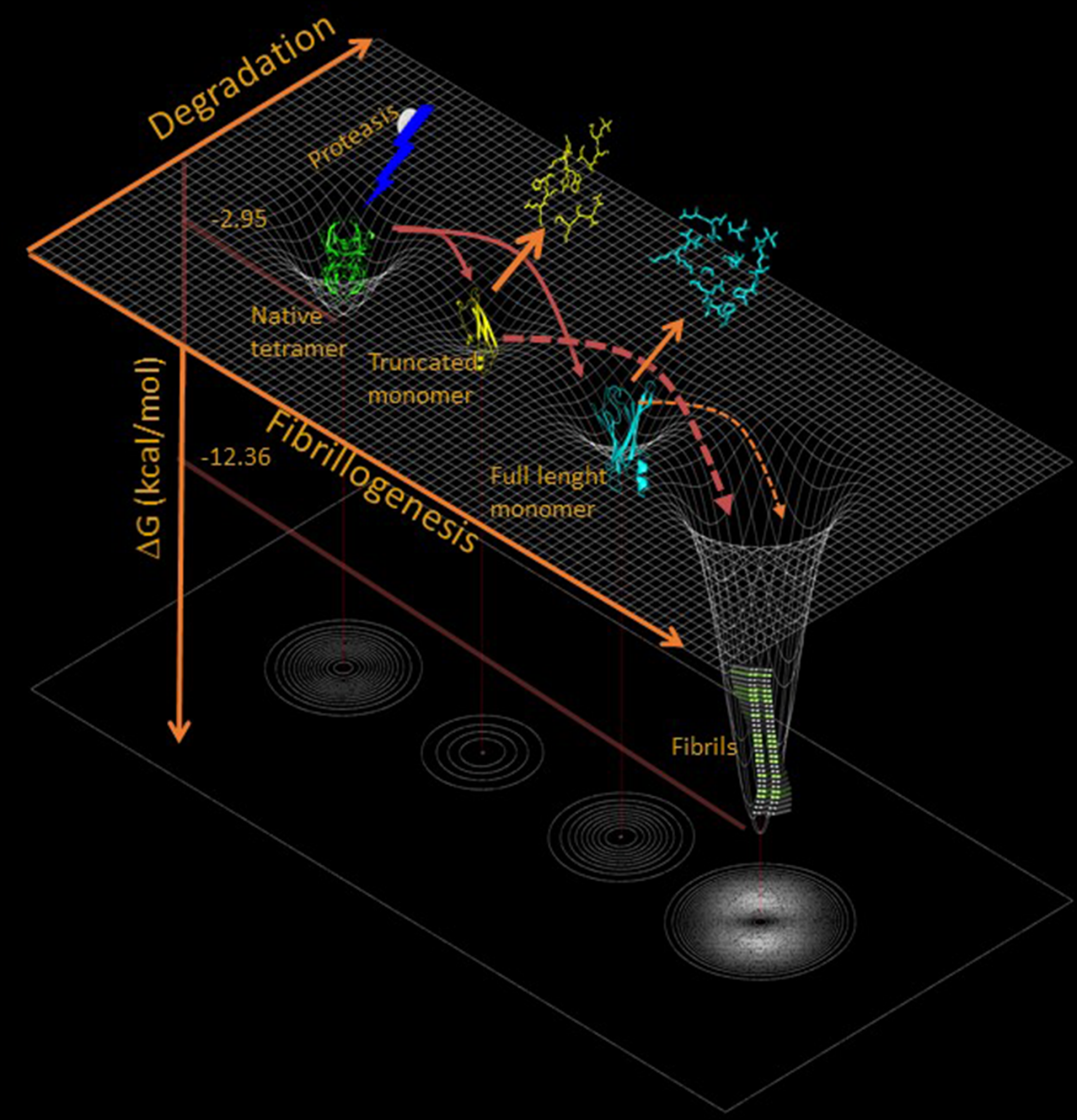
Mechano‐enzymatic mechanism of TTR fibrillogenesis. Cleavage of the native tetramer results in its dissociation into truncated and full‐length monomers, which are then further digested into smaller non‐aggregation‐prone peptides or become suitable for fibrillar nucleation. Image created with BioRender.com. TTR, transthyretin.

The involvement of a proteolytic event in the so‐called mechano‐enzymatic mechanism (Marcoux et al., 2015) implies a much more complex content of protein conformers. The selective cleavage at position Lys48‐Thr49, even in a single protomer out of the four assembled in the native TTR, causes the destabilization and disassembly of the tetramer. Both full‐length and truncated disassembled monomers are intrinsically highly susceptible to further proteolytic digestion because all the residues initially involved in the intermolecular/inter‐subunits interactions become exposed and highly dynamic in the aqueous solvent. Therefore, we can expect a heterogeneous population of conformers in which full‐length monomers coexist with two major fragments, one spanning residues 49–127 and the second corresponding to residues 81–127 together with many other peptides derived from the sub‐digestions of the major species (Figure 2). Degradation and fibrillogenesis become two alternative pathways diverging from the same proteolytic event. Upon cleavage, native TTR starts to be consumed and the amyloidogenic conformers cannot accumulate during the lag phase as they may be further digested into the degradation pathway. Therefore, gathering insight into the length of the lag phase is crucial since it is most likely directly proportional to the efficiency of the degradation pathway. Once the first nuclei of fibrils are formed and stabilized, a significant shift of the equilibrium between the two processes favoring fibrillogenesis over degradation can be predicted. Common constituents of the extra‐cellular matrix, such as glycosaminoglycans (GAGs), or amyloid seeds may play a role in shifting the equilibrium toward the formation of amyloid fibrils. The modulation of the lag phase by the addition of fibrillar seeds has been extensively shown and reviewed, but never explored in relation to the mechano‐enzymatic mechanism of fibril formation. Indeed, the role of fibrillar seeds in TTR amyloidogenesis is of particular interest. Several clinical studies strongly support the relevant role that preformed fibrils may have in the natural history of patients affected by familial forms of polyneuropathy, who received liver transplantation to abrogate the expression of the pathogenic TTR variant and later experienced disease recurrence due to the accumulation of wild‐type TTR on pre‐existing amyloid deposits (Ando et al., 1995; Holmgren et al., 1993). Nuclei of amyloid deposits may also influence the clinical response to new therapies targeting TTR, including protein stabilizers and inhibitors of TTR expression. In the presence of pre‐existing amyloid deposits, response to these therapies might require a specific level of stabilization for each variant and/or a different degree of suppression in order to obtain an optimal therapeutic effect. Therefore, we have investigated the effect of amyloid seeds, as initiators, and heparin, as surrogate for a generic physiological fibril‐stabilizer, on the fibrillogenesis of two TTR variants responsible for some of the most aggressive forms of cardiac amyloidosis, S52P (Stangou et al., 1998) and L111M TTR (Frederiksen et al., 1962).

Mechano‐enzymatic aggregation was carried out using both trypsin and plasmin, two proteases presenting the same specificity but different catalytic efficiency on TTR (Mangione et al., 2018). The use of the two proteases has revealed differences in the kinetic response by the two variants and a different modulation of amyloid formation by seeds and heparin. Our findings highlight the hypothesis that multiple biological factors, acting on each specific TTR variant, may influence the rate of amyloid growth *in vivo*.

## 2 RESULTS

### 2.1 Review of previously reported clinical data associated with L111M and S52P TTR variants

The pathogenic role of the L111M single‐mutation was first described by Frederiksen et al. (1962) in a large Danish kindred. Five out of 12 siblings developed progressive cardiac failure in their 40s due to a prominent TTR amyloid cardiomyopathy, which was clinically well‐documented.

The S52P mutation was first clinically described by Stangou et al. (1998) and, more recently, further characterized by González‐Duarte et al. (2013) and Mangione et al. (2014). This variant is always associated with early and severe cardiac involvement with dysautonomia symptoms, peripheral neuropathy, and ophthalmopathy.

### 2.2 Comparative analysis of thermodynamic stability of the two TTR variants

**FIGURE 3:**
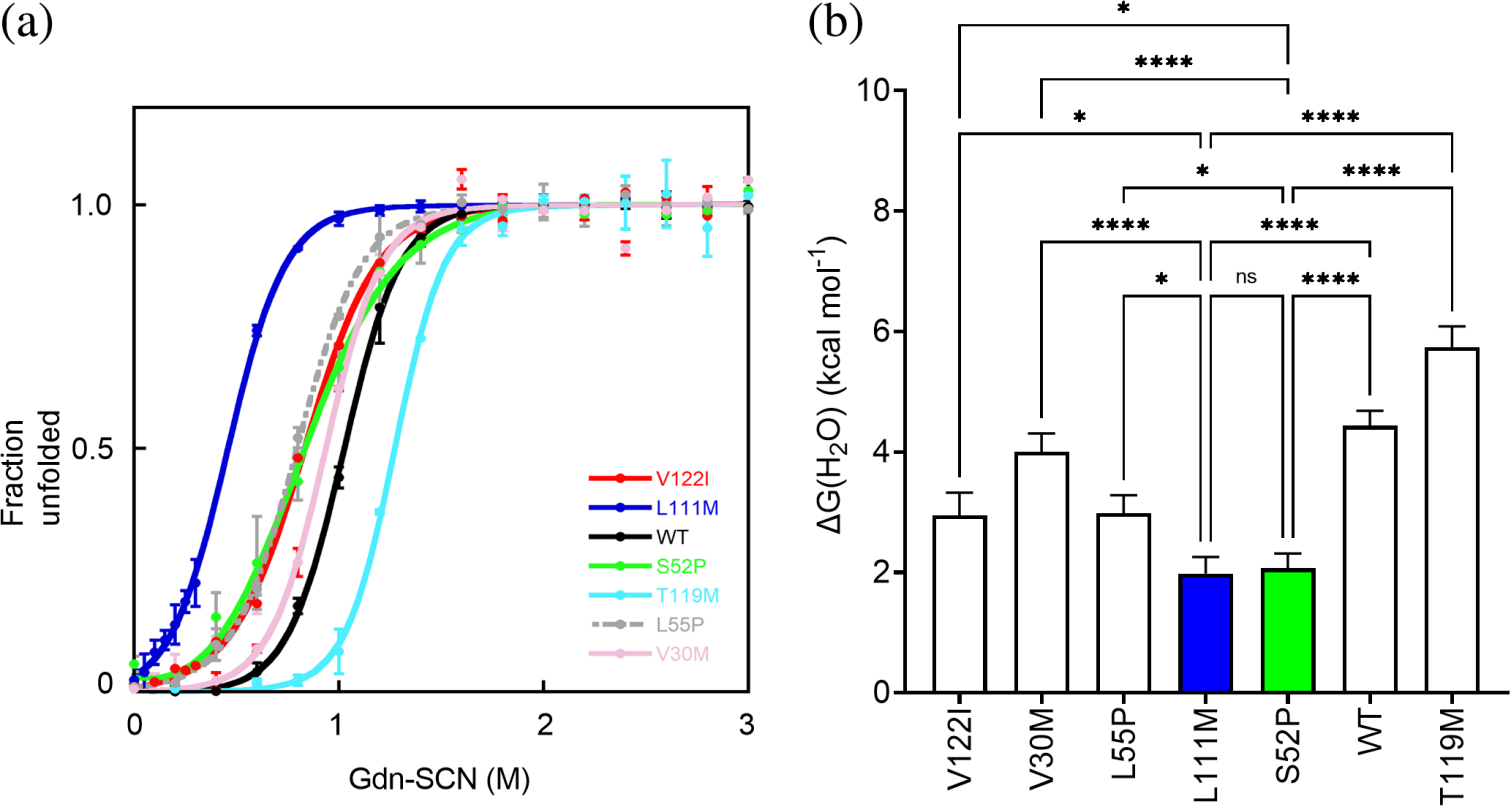
Thermodynamic stability of different TTR variants. (a) Denaturation profile for TTR variants derived from the change in fluorescence (F355/F335) following 295 nm excitation and calculated according to a two‐state model of unfolding (Santoro & Bolen, 1988). Curves shown as mean ± SD of at least three independent experiments. (b) Values of ΔG^(H2O)^ shown as mean ± SD of at least three independent experiments. Two‐way ANOVA test for multiple comparisons shown for L111M TTR (blue bar) or S52P TTR (green bar) and each other variant. ANOVA, analysis of variance; ns, non‐significant; SD, standard deviation; TTR, transthyretin. *p* < 0.05 (*), *p* < 0.005 (**), *p* < 0.0005 (***), *p* < 0.0001 (****).

We have studied the thermodynamic stability of both native S52P and L111M variants by classical guanidine thiocyanate (Gdn‐SCN) mediated transition from folded tetramer to unfolded monomers (Hammarstrom et al., 2001) as previously used to characterize S52P variant in comparison with wild‐type TTR (Mangione et al., 2014). The fraction of unfolded protein of L111M or S52P TTR was compared with other amyloidogenic variants (Figure 3a) in addition to wild‐type (Mangione et al., 2014) and V122I TTR (Raimondi et al., 2020) previously investigated. The ratio between the intrinsic tryptophan fluorescence intensities of the unfolded monomer at 355 nm and the folded tetramer at 335 nm were normalized from linear baseline dependence on denaturant and fitted to a two‐state unfolding transition (Santoro & Bolen, 1988). Values of energy of unfolding between the folded and unfolded state in the absence of denaturant (Δ*G* ^(H2O)^), the dependence on denaturant concentration (*m*), and midpoint denaturant concentration (*C*_*M*_), as mean and standard deviation (SD) of at least three independent experiments were extrapolated from the curves to evaluate the thermodynamic stability of TTR isoforms (Table 1). Pairwise multiple comparisons of Δ*G* ^(H2O)^ values between L111M TTR or S52P TTR and other TTR variants showed that both were significantly less stable than the others (Figure 3b).

**TABLE 1:** Thermodynamic parameters of Gdn‐SCN induced TTR unfolding.

TTR variant	^a^Δ*G* ^(H2O)^	^a^ *C*_*M*_	^a^ *m*
V122I	2.95 ± 0.4	0.8 ± 0.06	3.5 ± 0.4
V30M	4.00 ± 0.3	0.9 ± 0.01	4.3 ± 0.3
L55P	2.98 ± 0.3	0.8 ± 0.01	3.7 ± 0.3
L111M	2.00 ± 0.3	0.5 ± 0.02	4.2 ± 0.4
S52P	2.10 ± 0.2	0.8 ± 0.06	2.4 ± 0.2
WT	4.43 ± 0.3	1.0 ± 0.02	4.3 ± 0.3
T119M	5.74 ± 0.3	1.3 ± 0.01	4.5 ± 0.2

Abbreviations: SD, standard deviation; TTR, transthyretin.

^a^ Values of free energy of unfolding in the absence of denaturant, ΔG^(H2O)^ (kcal mol^−1^) and dependence of Δ*G* on denaturant concentration, *m* (kcal mol^−1^ M^−1^) were directly extrapolated from equilibrium denaturation curves of all globular TTR variants using a two‐state model (Santoro & Bolen, 1988). Midpoint concentrations of Gdn‐SCN, *C*_*M*_ (M), were determined as Δ*G* ^(H2O)^/m. All values are reported as mean ± SD of at least three independent experiments.

### 2.3 Limited proteolysis of S52P and L111M TTR

**FIGURE 4:**
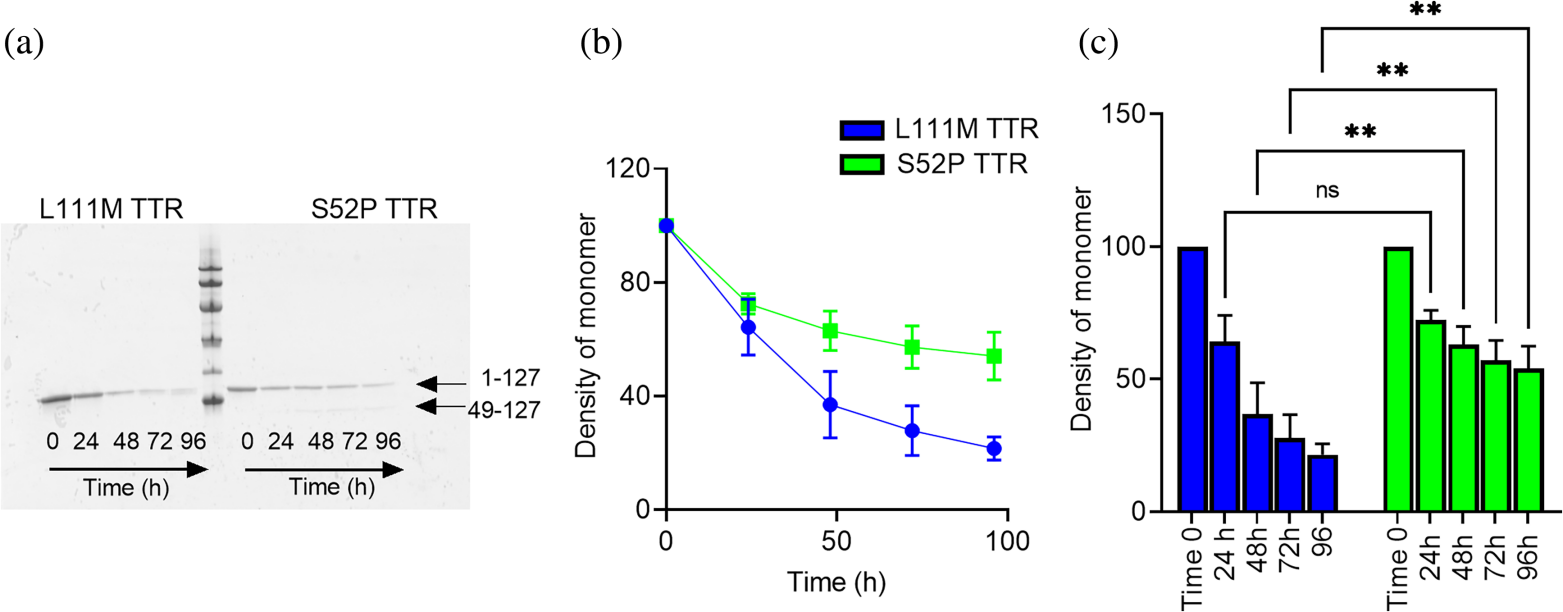
Limited proteolysis of S52P and L111M TTR. (a) Representative homogeneous 15% SDS‐PAGE of L111M and S52P TTR samples at different time points during static digestion (0, 24, 48, 72, and 96 h, respectively). Marker proteins (14.4, 20.1, 30.0, 45.0, 66.0, and 97.0 kDa). (b) Decrease in TTR monomer band intensity over time following trypsin digestion quantified using the Quantity One software (Bio‐Rad). Intensities of the electrophoretic bands corresponding to the intact protomer in the whole mixture were normalized to 100% for the same band of the protein before addition of trypsin. Each data point is mean ± SD of three independent experiments with solid line as a guide to the eye for S52P (green) and L111M (blue) TTR, respectively. (c) A two‐way ANOVA test for multiple comparisons shown to highlight differences in kinetics of monomer consumption with time. ANOVA, analysis of variance; ns, non‐significant; SD, standard deviation; TTR, transthyretin. *p* < 0.005 (**).

Our previous work has shown that S52P TTR is highly susceptible to proteolysis at very low substrate:enzyme ratio in the absence of agitation, unlike other variants including wild‐type, V122I, V30M, and L55P (Mangione et al., 2014). Given the low thermodynamic stability determined for L111M TTR, we hypothesized that this highly destabilized variant could also be susceptible to proteolysis in static conditions. L111M TTR was indeed proteolyzed with trypsin at 37°C without agitation. Monomer digestion and formation of amyloidogenic fragments were both monitored over time in SDS‐PAGE electrophoresis (Figure 4a). The intensity of the gel band corresponding to monomeric TTR decreased over time for both proteins (Figure 4b) with a significantly faster decay for L111M TTR than for the S52P variant at 48, 72, and 96 h, respectively (Figure 4c).

### 2.4 Prediction of the 49–127 TTR fragment aggregation propensity

**FIGURE 5:**
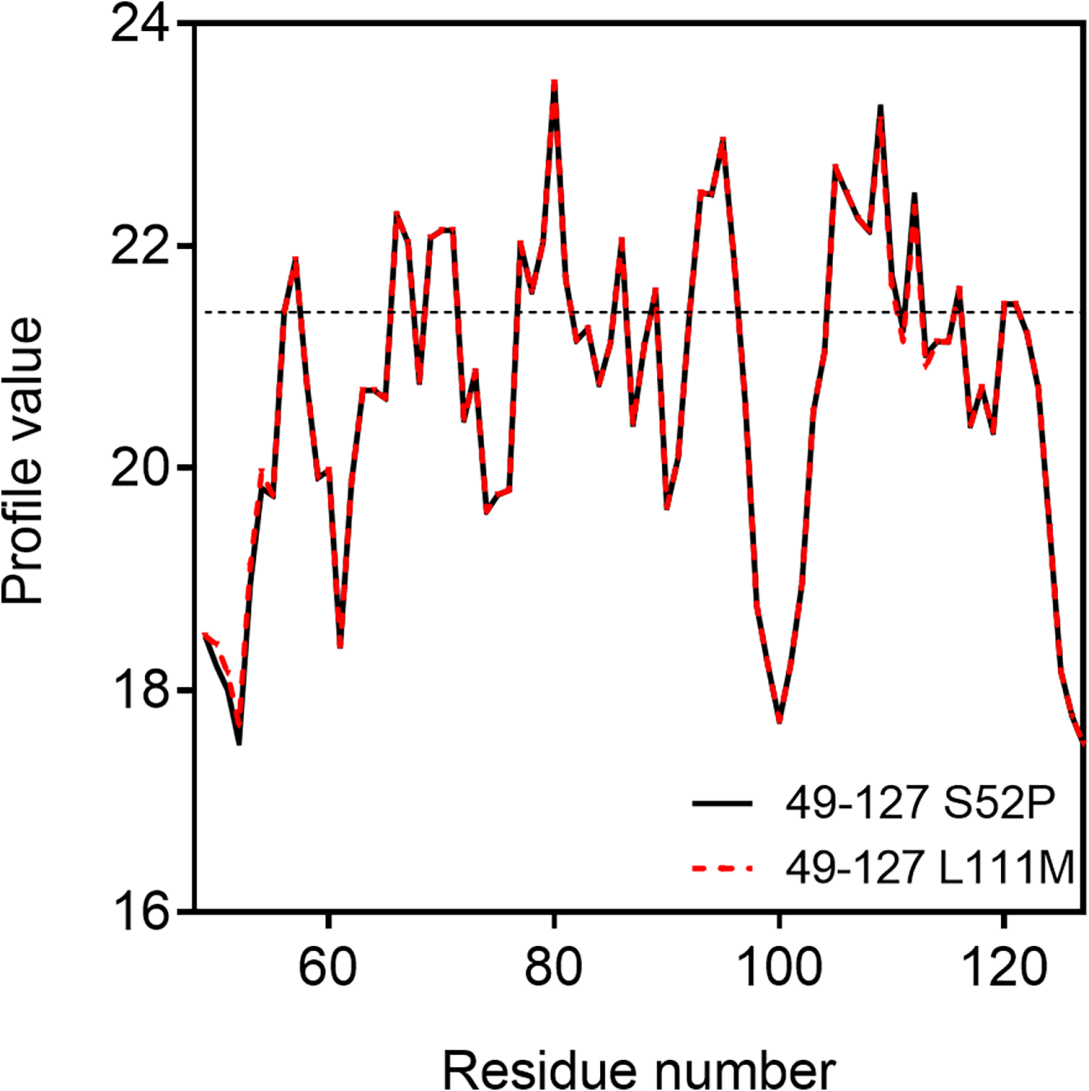
Prediction of aggregation propensity by FoldAmyloid. Aggregation propensity of the 49–127 TTR fragment generated by proteolytic cleavage of S52P (black) and L111M (red) TTR. TTR, transthyretin.

Mechano‐enzymatic cleavage of the two variants generates the aggregation‐prone 49–127 TTR fragment, whose sequence differs between the two variants given that the specific mutations, in positions 52 and 111, respectively, are located within the amyloidogenic fragment itself. The online protein aggregation propensity software FoldAmyloid (Pinheiro et al., 2021) predicted no major differences as a result of the two distinct single amino acid substitutions on the overall aggregation propensity of the 49–127 TTR fragment (Figure 5).

### 2.5 Comparative fibrillogenesis of the two variants and effect of heparin

**FIGURE 6:**
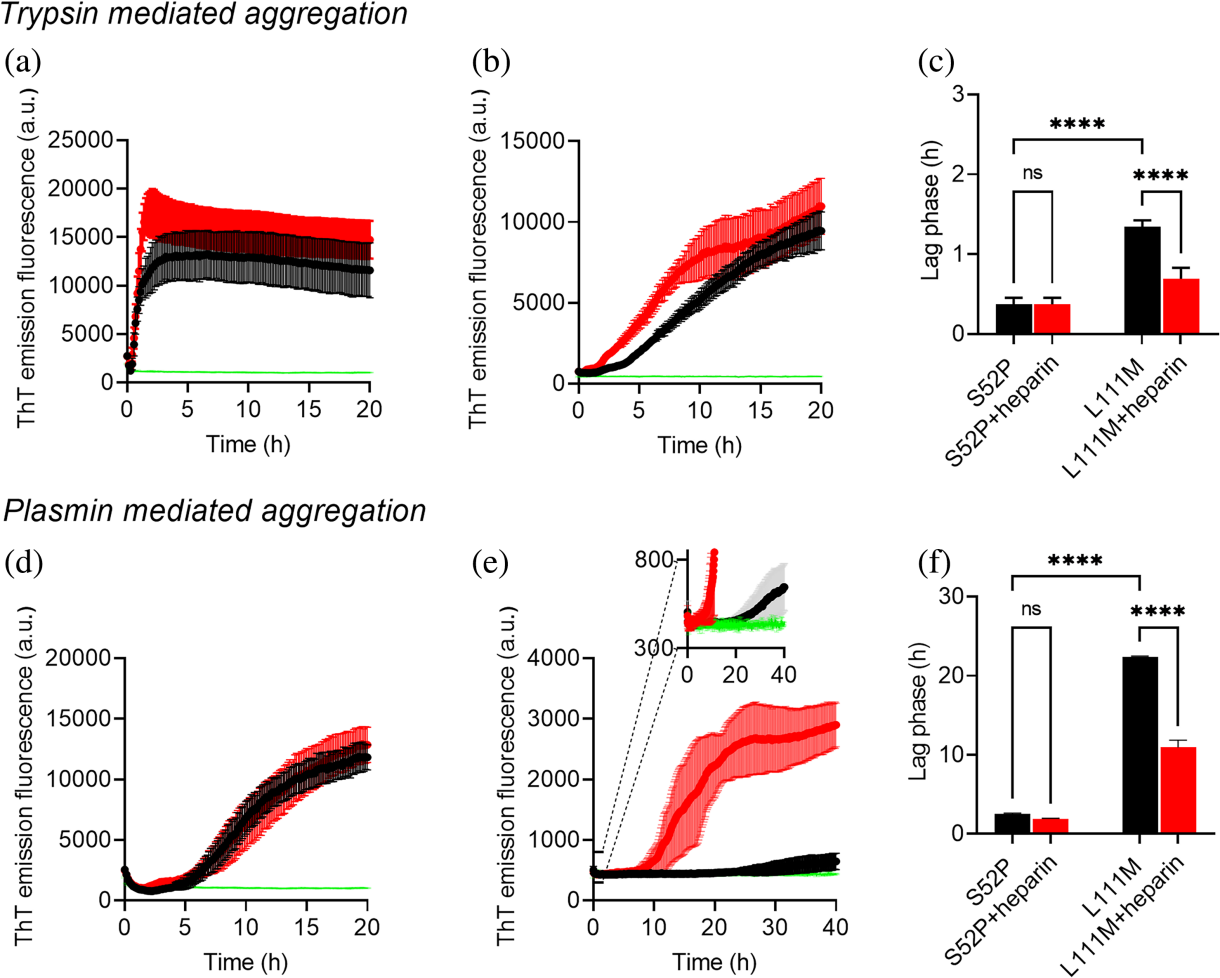
Effect of heparin on S52P and L111M TTR aggregation. (a) S52P TTR and (b) L111M TTR trypsin‐mediated aggregation monitored by ThT emission in the absence (black) and presence (red) of 12 μM heparin. (c) Lag phases of trypsin‐mediated fibril formation by S52P and L111M TTR in the absence and presence of heparin. (d) S52P TTR and (e) L111M TTR plasmin‐mediated aggregation monitored by ThT emission in the absence (black) and presence (red) of 12 μM heparin. Inset: expanded view of the ThT signal between 300 and 800 a.u. to highlight differences with control experiment in which L111M TTR is incubated in the absence of plasmin (green). (f) Lag phases of plasmin‐mediated fibril formation of S52P and L111M TTR in the absence and presence of heparin. Control experiments without the corresponding enzyme are shown for each variant (green). All curves shown as mean ± SD of three independent experiments. Values of lag phases are extrapolated from each curve at the time where the signal relative to the baseline has reached 10% of the amplitude of the ThT emission fluorescence increase (Arosio et al., 2015). A two‐way ANOVA test for multiple comparisons shown to highlight differences in lag phases for corresponding fibril formation by S52P and L111M TTR. ANOVA, analysis of variance; ns, non‐significant; SD, standard deviation; TTR, transthyretin. *p* < 0.0001 (****).

The increase in ThT emission fluorescence monitored during incubation of each protein in the presence of trypsin and double orbital shaking (Figure 6a,b) revealed a significant difference in the kinetics of fibrillogenesis of the two variants (Figure 6c) with the lag phase (all shown as mean ± SD, *n* = 3) of S52P variant (0.37 ± 0.08 h) shorter than that of L111M TTR (1.4 ± 0.08 h). When the mechano‐enzymatic fibrillogenesis was conducted in the presence of plasmin, the rate of fibril formation of both variants was significantly reduced in comparison with the trypsin‐mediated process (Figure 6d,e). The lag phase for the aggregation in the presence of plasmin increased to 2.50 ± 0.14 h for S52P TTR compared with the same parameter determined for the fibrillogenesis carried out in the presence of trypsin (*p* < 0.0001). At the same time, the lag phase for the plasmin‐mediated process increased to 22.41 ± 0.08 h (mean ± SD, *n* = 3) for L111M TTR compared with the value determined for the fibril formation performed with trypsin (*p* < 0.0001). In the presence of plasmin, not only L111M TTR aggregate significantly more slowly than S52P TTR (Figure 6f), but also the yield in amyloid fibrils as related to the ThT emission fluorescence appeared reduced in comparison with the yield of L111M TTR fibrils in the presence of trypsin (Figure 6b,e).

In the context of different modulators of fibrillogenesis, it is widely accepted that GAGs can have an important role on amyloid formation *in vivo*. GAGs, such as heparin and several analogs of heparans, are common constituents of all natural amyloid plaques (Snow et al., 1987). They can accelerate, *in vitro*, the fibrillogenesis of several amyloidogenic proteins through specific interactions recently thoroughly investigated by Khurshid et al. (2022). A pro‐aggregating role for heparin was also previously identified in acidic‐mediated TTR aggregation, mainly due to a selective binding to the 24–35 domains of TTR (Noborn et al., 2011). Here, we have studied the effect of heparin on the mechano‐enzymatic fibrillogenesis of the two variants exposed to trypsin (Figure 6a–c) or plasmin (Figure 6d–f) at physiological pH, ionic strength, and temperature.

Interestingly, we found that heparin had no effect on the extremely fast kinetics of S52P TTR in the presence of trypsin (Figure 6a,c). Similarly, there was non‐significant difference when plasmin was used in the fibrillogenesis of the same variant, when the lag phase was longer than in the presence of trypsin (Figure 6d,f).

On the contrary, heparin contributed to modify the kinetics of fibril formation of the L111M TTR variant both in the presence of trypsin, with a lag phase of 0.69 ± 0.14 h (Figure 6b,c) and in the presence of plasmin (Figure 6e,f) in which the lag phase was reduced to 10.92 ± 0.92.

### 2.6 Effect of amyloid seeds on TTR fibrillogenesis

**FIGURE 7:**
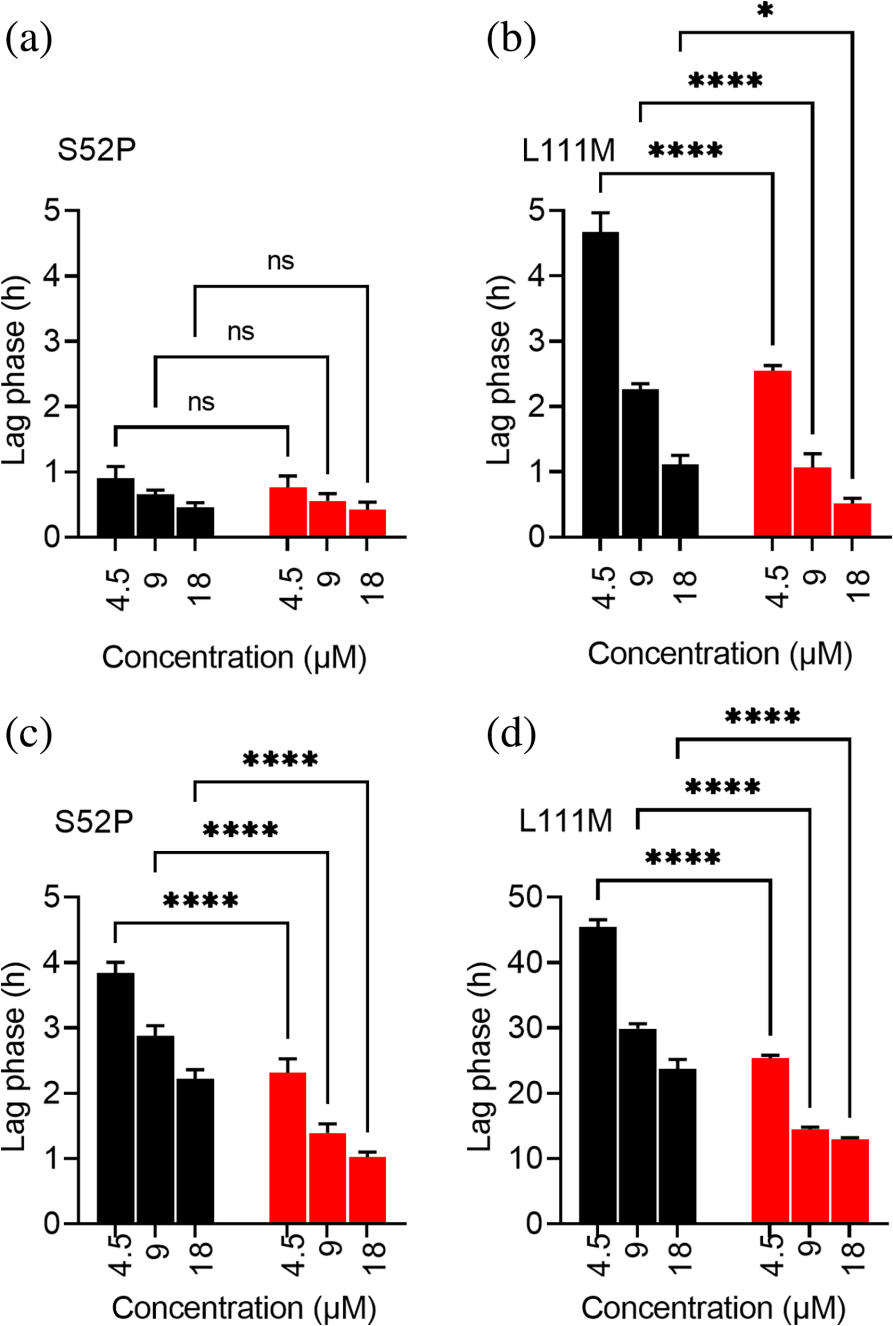
Effect of seeds on S52P and L111M TTR aggregation. (a) S52P TTR and (b) L111M TTR lag phases for seeded (red) and unseeded (black) samples at different protein concentrations in the presence of trypsin. (c) S52P TTR and (d) L111M TTR lag phases for seeded (red) and unseeded (black) samples at different protein concentrations in the presence of plasmin. Experimental data shown as mean ± SD of three independent experiments. A two‐way ANOVA test for multiple comparisons shown to highlight the effect of seeds for each protein concentration. ANOVA, analysis of variance; ns, non‐significant; SD, standard deviation; TTR, transthyretin; *p* < 0.05 (*), *p* < 0.0001 (****).

Amyloid seeds are known to minimize the lag phase preceding the aggregation of amyloidogenic proteins (Saelices et al., 2018). We have investigated the effect of pre‐formed nuclei on the mechano‐enzymatic fibrillogenesis of S52P and L111M TTR in the presence of trypsin or plasmin, by measuring the lag phase of the process at different concentrations of TTR and constant total amount of the added seeds. The kinetics of TTR mechano‐enzymatic fibrillogenesis inversely correlated with protein concentration for both variants, as shown in Figure 7, in which the length of the lag phase of fibril formation, in the presence and absence of seeds, was plotted against the corresponding TTR concentration exposed to trypsin (Figure 7a,b) or plasmin (Figure 7c,d). A thorough analysis of the data showed how seeds affected differently the fibrillogenesis of the two variants in relation, also, to the specific protease in use. No significant change was indeed observed when seeds were added to S52P TTR exposed to trypsin (Figure 7a) compared to the acceleration induced when the same variant was digested with plasmin. Statistical analysis showed that plasmin‐mediated fibrillogenesis of S52P TTR was significantly accelerated, with a *p* value <0.0001 at all protein concentrations here analyzed (Figure 7c). On the other hand, the addition of seeds to L111M TTR significantly reduced the lag phase of fibril formation both in the presence of trypsin (Figure 7b) as well as of plasmin (Figure 7d).

## 3 DISCUSSION

The transition of globular proteins into amyloid fibrils, occurring in amyloid diseases, is still a largely unexplained biological process. We have hypothesized (Marcoux et al., 2015) that, particularly in TTR, a specific proteolytic event destabilizes the folded state and trigger the amyloid transformation of the globular protein into fibrils. The combination of biomechanical forces and proteolytic activity, which occurs in cardiac TTR amyloidogenesis, fits with the concept of a mechano‐enzymatic mechanism as a pathological process, which is also well described for a relevant physiological process unrelated to amyloid diseases (Zhang et al., 2009).

The scheme presented in Figure 2 (Raimondi et al., 2020) highlights the complexity of the amyloid pathway of a globular protein when proteases are involved in the process. The experimental dissection of the mechano‐enzymatic mechanism is extremely challenging and further studies would need to accurately characterize the formation and/or degradation of the proteolytic fragments. We have assumed that limited and selective proteolytic events highly destabilize the quaternary structure of the protein with the release of both truncated and full‐length monomers. Misfolded monomers can be further digested or, generate amyloid fibrils. Therefore, degradation and fibrillogenesis become two alternative pathways for a misfolded conformer. Equilibrium between the two processes can deeply influence the yield of fibrils *in vitro*, whereas *in vivo*, it could modulate the natural history of the disease.

We have studied the mechano‐enzymatic fibrillogenesis of two highly unstable TTR variants aiming to evaluate whether the scheme in Figure 2 is consistent with the results obtained *in vitro* at different concentrations and in the presence or absence of modulators of fibrillogenesis.

We have previously shown (Mangione et al., 2018) that two proteases, trypsin and plasmin, have the same specificity to cleave native TTR, but different kinetics, with trypsin being more efficient than plasmin.

Among other well‐known amyloidogenic variants, S52P and L111M TTR are the most thermodynamically unstable. Clinical reports of a few families carrying the two mutations suggest that the heart is an early and major target for both, although with a high degree of heterogeneity in onset, rate of progression, and involvement of other organs (Frederiksen et al., 1962; González‐Duarte et al., 2013; Stangou et al., 1998).

The two variants, despite a similar level of destabilization, based on their value of free energy between folded and unfolded states, have a very different susceptibility to proteolytic digestion, thus highlighting that the topological dynamics at the site of cleavage may be more important than the overall stability of the tetramer. L111M TTR seems to be more susceptible than the S52P variant to trypsin digestion, although the effect of proteolysis on fibrillogenesis is the opposite. These data can be interpreted, according to the scheme in Figure 2, as a condition in which the degradative process prevails over fibrillogenesis; therefore, TTR proteolytic fragments cannot proceed to amyloid formation. Based on the experiments shown here, L111M TTR would be interpreted much less aggressive than the S52P variant. However, the scenario can change in the presence of heparin and its analogs, which are common constituents of natural amyloid fibrils and physiologically present in the extra‐cellular matrix of each organ. Heparin can modify the kinetics of fibril formation of the L111M variant in terms of both yield and reduction of the lag phase. This finding suggests that heparin favors fibrillogenesis as it may recruit those TTR conformers populating the lag phase of the process that would not otherwise reach the critical concentration required for the formation of fibrils, and, therefore, would be further digested by the proteases.

The addition of fibrillar seeds has a similar effect as heparin on the kinetics of fibrillogenesis of the two variants. The presence of seeds can hardly affect fibrillogenesis when it is very fast as it occurs for S52P TTR in the presence of trypsin, but becomes particularly relevant for plasmin‐mediated aggregation pathway of L111M TTR where seeds can induce the formation of fibrils even at low TTR concentration.

In conclusion, our experiments have further highlighted that the structural effect of single mutations can only partially explain the amyloid formation by TTR. In the biological environment, destabilizing mutations can promote degradation without the formation of amyloid fibrils. Nevertheless, modifications of the environment, such as local overexpression of GAGs, can significantly change the equilibrium between degradative and fibrillogenic pathways promoting the latter. Finally, we have shown that the presence of fibrillar seeds can significantly change the equilibrium between degradative proteolysis and fibrillogenic pathways. The mechano‐enzymatic model of TTR fibrillogenesis, in comparison to other models of fibrillogenesis, does not require a non‐physiological destabilization of the TTR tetramer; therefore, we are in conditions mimicking the conformation of circulating TTR at the time of interaction with specific proteases. All the polypeptides resulting from the first proteolytic cleavage and further sub‐digestion are potential intermediates of both degradative and fibrillogenic pathways. We have also shown the influence of heparin as one of the putative modulators of the kinetics of the two pathways. Many other molecular entities can play a role in shifting the equilibrium between the two pathways and we believe that our observations may promote further studies to address the actual complexity of TTR amyloidogenesis in a biological environment.

## 4 MATERIALS AND METHODS

### 4.1 Protein expression and purification

Transformed BL21 star (DE3) cells (Thermo Fisher Scientific), containing the peTM11 plasmid encoding hexahistidine‐tagged S52P or L111M TTR, were plated onto Luria broth (LB)‐agar media containing 30 μg/mL kanamycin for overnight incubation at 37°C. A single colony was isolated and cultured overnight at 37°C in a 5 mL LB medium containing 30 μg/mL kanamycin under shaking conditions (LB/kan). This preparation was inoculated into 1 L LB/kan for an initial growth at 37°C. When the culture reached OD_600_ = 0.5, the temperature was reduced to 30°C. Protein expression was induced at OD_600_ = 0.6 by adding IPTG (1 mM final concentration) for overnight incubation. The cells were harvested by centrifugation at 3500 *g*, suspended in lysis buffer containing 20 mM Tris–HCl pH 8, 250 mM NaCl, 3 mM imidazole, and finally sonicated at 4°C. The supernatant was clarified following 30 min centrifugation at 18,000 *g* and loaded onto a HisTrap FF crude nickel affinity chromatography column (GE Healthcare) equilibrated in lysis buffer. After extensive washing with 20 mM Tris–HCl, 10 mM imidazole, containing stepwise increasing concentrations of NaCl (250 mM, 500 mM, and 1 M, respectively), the column was eluted with 20 mM Tris–HCl, 250 mM NaCl, and 250 mM imidazole, pH 8.0. His‐tagged TEV protease (Sigma‐Aldrich) was added at 1% w/w during dialysis to selectively cleave the hexaHistidine‐tag, which was then removed by affinity chromatography, together with the enzyme. Fractions containing TTR were pooled and subjected to size exclusion chromatography using a Superdex 75 Hi Load 26/60 column (GE Healthcare) equilibrated and eluted with 25 mM Tris–HCl, 100 mM NaCl, pH 8.0. Fractions containing TTR were dialyzed against water at 4°C for at least 3 days and then lyophilized. Purity and molecular weight were confirmed by SDS‐PAGE analysis and mass spectrometry, respectively.

### 4.2 Equilibrium unfolding of TTR variants

Samples containing native recombinant human TTR (0.1 mg/mL, corresponding to 1.8 μM tetramer) were incubated at increasing concentrations of Gdn‐SCN in 50 mM phosphate, 1 mM EDTA, 1 mM DTT, pH 7.0, at 25°C for 24 h. Measurements were carried out in a 1 cm light path quartz cell using a PerkinElmer LS55 spectrofluorimeter and tryptophan emission spectra were recorded in the range between 310 and 400 nm after excitation of protein samples at 295 nm. Unfolding curves as function of Gdn‐SCN concentration were generated using the ratio between the intrinsic fluorescence intensities of the unfolded protein at 355 nm and the folded tetramer at 335 nm. Experimental data were fitted using the two‐state model equation of Santoro and Bolen (1988) to estimate the free energy of unfolding in the absence of denaturant (Δ*G* ^H2O^), the dependence of Δ*G* on denaturant concentration (*m* value) as previously described (Mangione et al., 2014; Raimondi et al., 2020). Midpoint Gdn‐SCN concentrations for each TTR variant were determined as Δ*G* ^(H2O)^/*m*.

To compare the stability of different TTR variants, experiment data were reported as apparent unfolded fractions using the formula:

$$\text{Fraction unfolded}=\left(y-y_{N}\right)/\left(y_{U}-y_{N}\right),$$

where *y* is the experimental value observed at a given denaturant concentration; *y*_*N*_ and *y*_*U*_ are the values of the native and unfolded protein, respectively, extrapolated from the pre‐ and post‐transition baselines defined by the two‐state model (Santoro & Bolen, 1988). Experimental data were fitted using Kaleidagraph 4.0 (Synergy Software, Reading, PA, USA). A two‐way analysis of variance (ANOVA) followed by Tukey's pairwise multiple comparisons test was carried out between L111M or S52P TTR and each other variant with GraphPad Prism 9.

### 4.3 Limited proteolysis of S52P and L111M TTR

Recombinant S52P and L111M variant TTR were individually digested at 1 mg/mL in phosphate‐buffered saline (PBS) (pH 7.4) at 37°C containing trypsin (Promega, V5280) at an enzyme: substrate (w/w) ratio of 1:200 under static conditions. At different time points, aliquots of each incubation mixture were boiled immediately in SDS sample buffer, and were stored at −20°C before SDS 15% PAGE was carried out under reducing conditions. A two‐way ANOVA test was performed using GraphPad Prism 9 for pairwise multiple comparisons to highlight the difference in kinetics of monomer digestion of the two variants.

### 4.4 Prediction of the 49–127 TTR fragment aggregation propensity

The aggregation propensity for both fragments was predicted using the online protein aggregation propensity software FoldAmyloid (http://bioinfo.protres.ru/fold-amyloid/) modifying the following 49–127 wild‐type TTR sequence.

TS ESGELHGLTT EEEFVEGIYK VEIDTKSYWK ALGISPFHEH AEVVFTANDS GPRRYTIAAL LSPYSYSTTA VVTNPKE

Replacing S in position 52 (highlighted in bold) with P for S52P TTR and, L in position 111 (underlined) with M for L111M variant, respectively.

### 4.5 TTR proteolysis under agitation

Recombinant S52P or L111M TTR, 100 μL volumes at 1 mg/mL in PBS, pH 7.4 was incubated at 37°C in Costar 96‐well black plates in the presence of a protease/substrate ratio of 5 ng/μL trypsin to yield a final enzyme:TTR ratio of 1:200. The plate was sealed with clear sealing film and subjected to 900 rpm double‐orbital shaking for 24 h. At different time points, aliquots of each incubation mixture were boiled immediately in SDS sample buffer, and were stored at −20°C before SDS 15% PAGE (Bio‐Rad) was carried out under reducing conditions using a Bio‐Rad gel apparatus.

### 4.6 TTR aggregation in the presence of heparin

Recombinant S52P or L111M TTR, 100 μL volumes at 1 mg/mL in PBS, pH 7.4 containing 10 μM ThT (Sigma‐Aldrich) in PBS and 12 μM heparin (Alfa‐Aesar) was incubated at 37°C in Costar 96‐well black plates (Corning Incorporated) in the presence of a protease/substrate ratio of 5 ng/μL trypsin (Promega, V5280) and 20 ng/μL plasmin (Sigma‐Aldrich, P1867) to yield a final enzyme:TTR ratio of 1:200 and 1:50, respectively. Control experiments with TTR variants incubated without enzyme were carried out in parallel. The plate was sealed with clear sealing film and subjected to 900 rpm double‐orbital shaking until the ThT signal reached a plateau. Bottom fluorescence was recorded at 500 s intervals (BMG LABTECH FLUOstar Omega). A two‐way ANOVA test was performed using GraphPad Prism 9 for pairwise multiple comparisons.

### 4.7 Preparation of amyloid seeds *in vitro*

Proteolysis‐mediated fibrillogenesis of both S52P and L111M TTR was carried out in glass vials (air/buffer interface of 1.5 cm^2^) stirred at 1500 rpm (IKA magnetic stirrer) and 37°C for 72 h using 1 mg/mL TTR in PBS, pH 7.4, in the presence of either 20 ng/μL plasmin (Sigma‐Aldrich, P1867) or 5 ng/μL trypsin. Fibrillar aggregates separated after a 20 min centrifugation at 10,300 *g* were quantified at the end of each procedure by the bicinchoninic acid (BCA) assay.

### 4.8 TTR aggregation in the presence of seeds

Recombinant S52P or L111M TTR, 100 μL volumes at different concentrations (1, 0.5, and 0.25 mg/mL corresponding to 18, 9, and 4.5 μM TTR tetramer, respectively) in PBS, pH 7.4 containing 10 μM ThT and 10 μg seeds was incubated at 37°C in Costar 96‐well black plates in the presence of a protease/substrate ratio of 1:200 for trypsin and 1:50 for plasmin. Depending on the specific proteolytic enzyme used in each experiment, the appropriate seeds prepared using the same enzyme were selected. The plate was sealed with clear sealing film and subjected to 900 rpm double‐orbital shaking until the ThT signal reached a plateau. Bottom fluorescence was recorded at 500 s intervals (BMG LABTECH FLUOstar Omega). A two‐way ANOVA test was performed using GraphPad Prism 9 for pairwise multiple comparisons.

## AUTHOR CONTRIBUTIONS

**Guglielmo Verona:** Investigation; methodology; data curation; funding acquisition; writing – original draft. **Sara Raimondi:** Investigation; methodology. **Diana Canetti:** Investigation; methodology. **P. Patrizia Mangione:** Investigation; data curation; writing – review and editing; formal analysis. **Loredana Marchese:** Investigation; methodology. **Alessandra Corazza:** Investigation; data curation. **Francesca Lavatelli:** Investigation; funding acquisition; writing – review and editing. **Julian D. Gillmore:** Investigation. **Graham W. Taylor:** Investigation; writing – review and editing. **Vittorio Bellotti:** Investigation; funding acquisition; conceptualization; writing – original draft. **Sofia Giorgetti:** Investigation; funding acquisition; writing – review and editing; conceptualization; supervision.

## CONFLICT OF INTEREST STATEMENT

The authors declare no conflict of interest.

## Data Availability

All data that support the findings of this study are available from the corresponding authors upon request.
